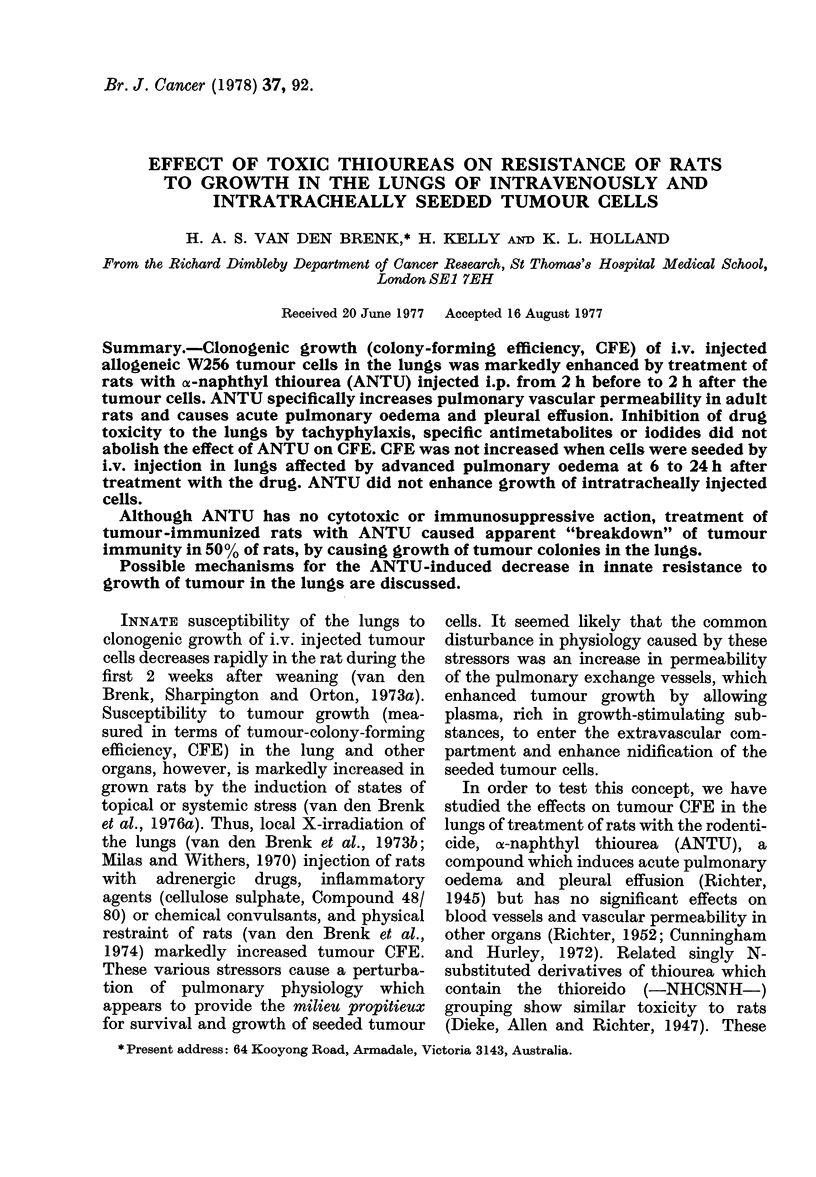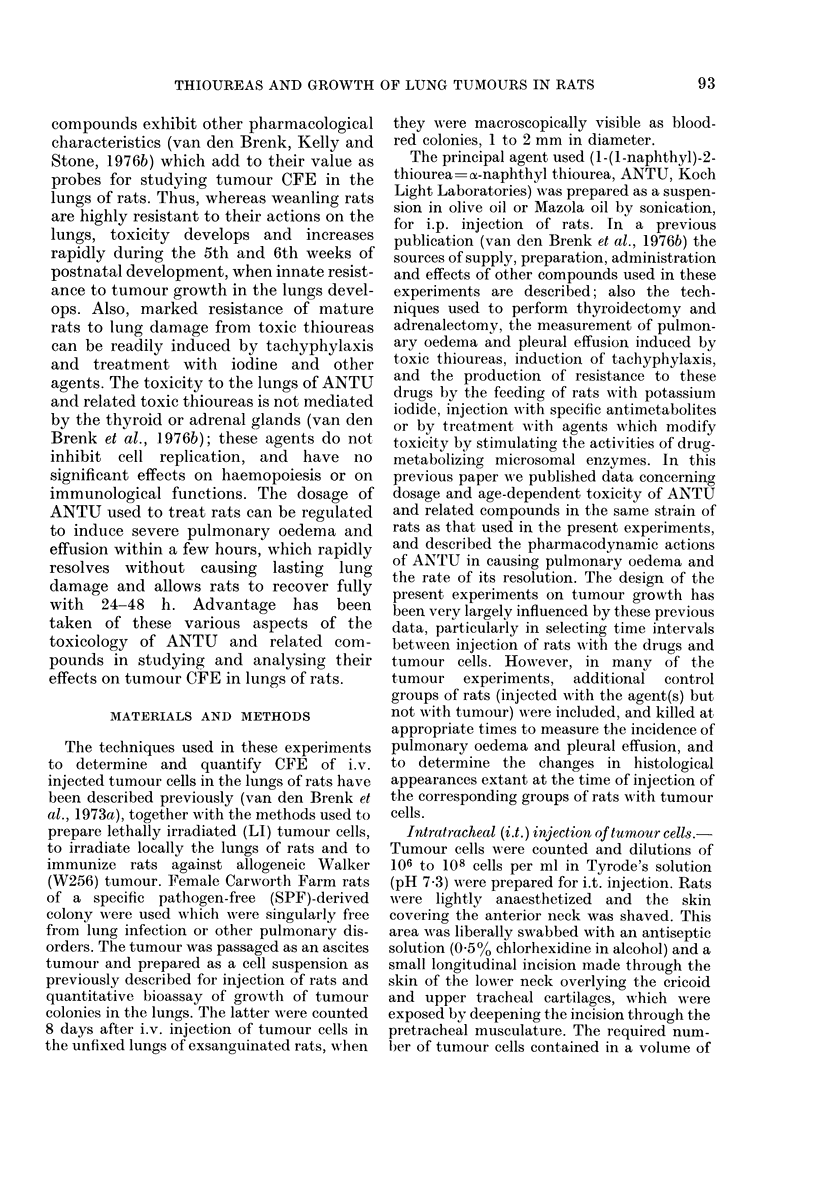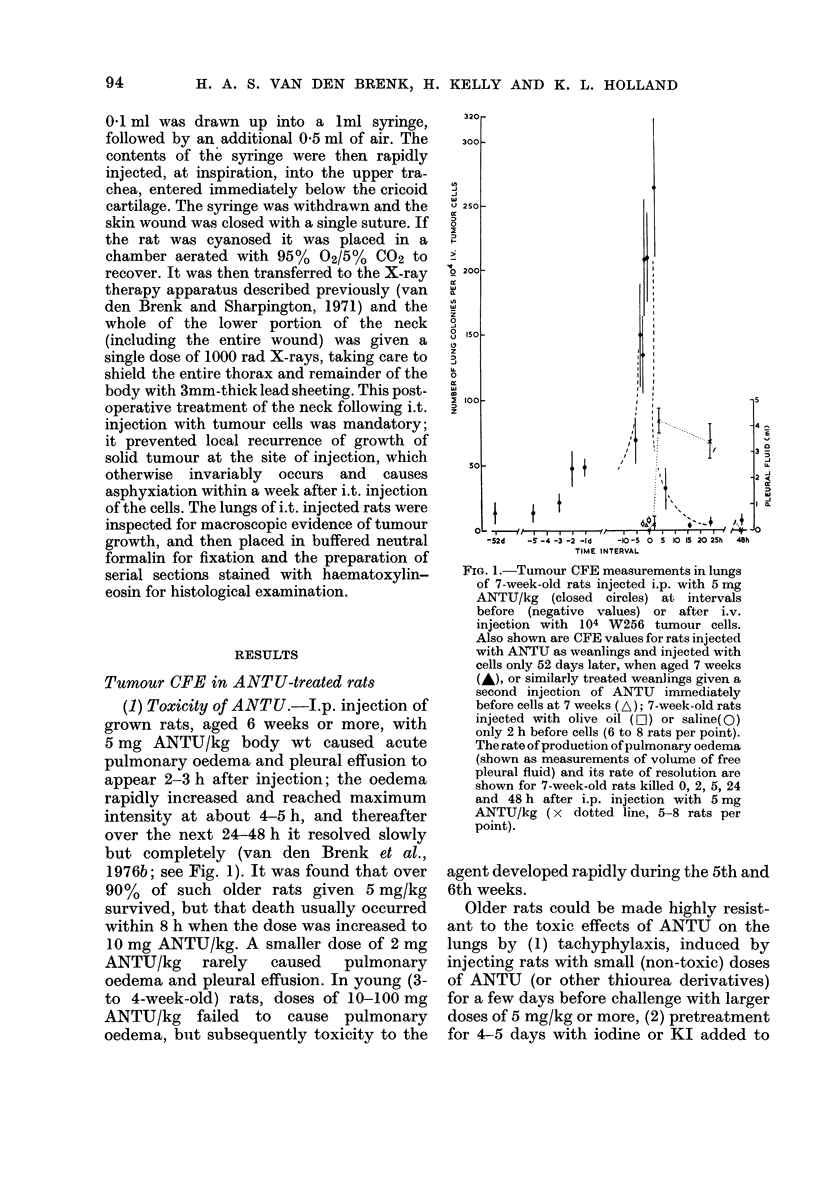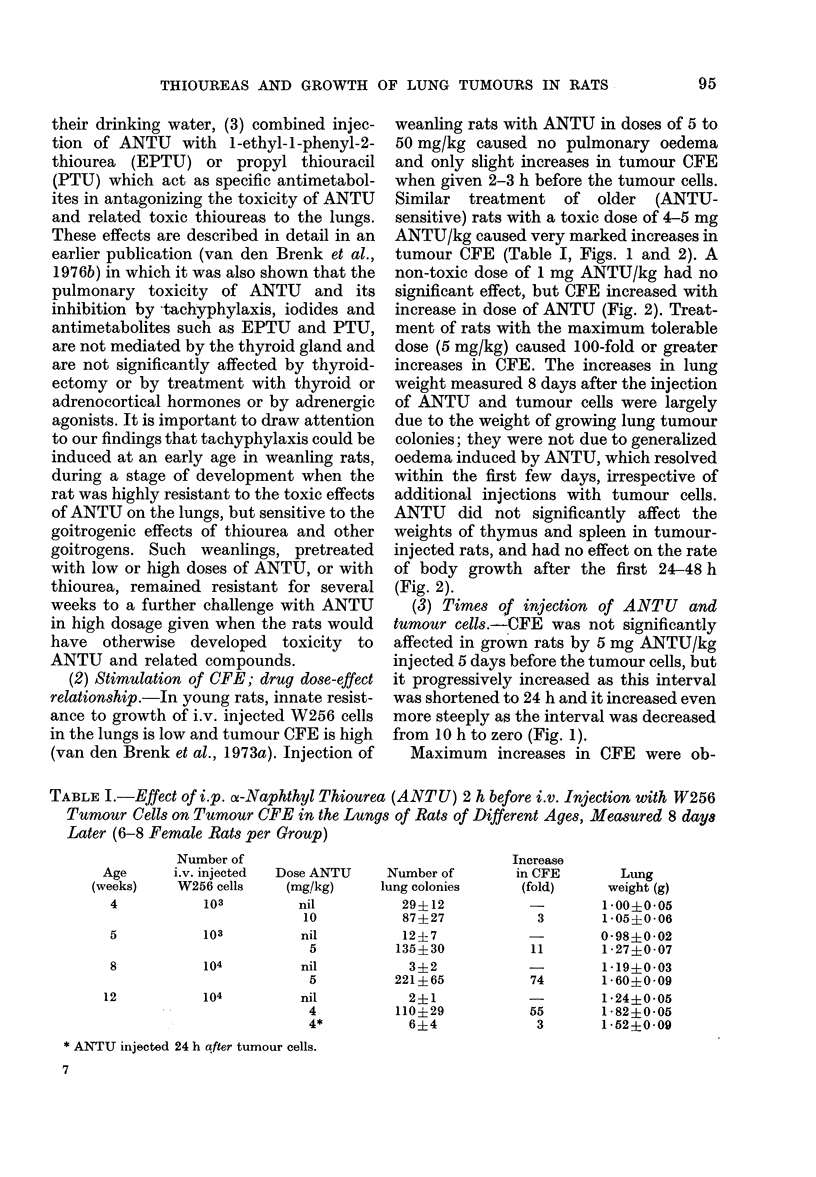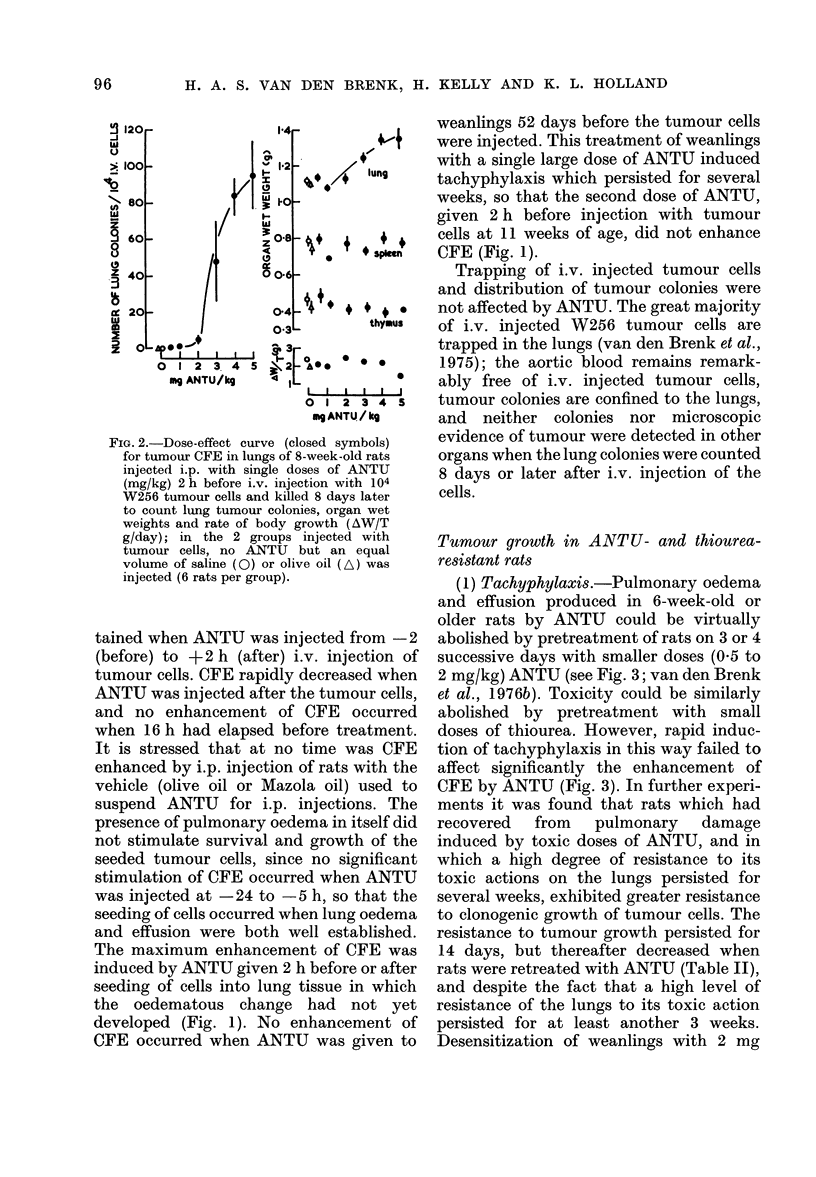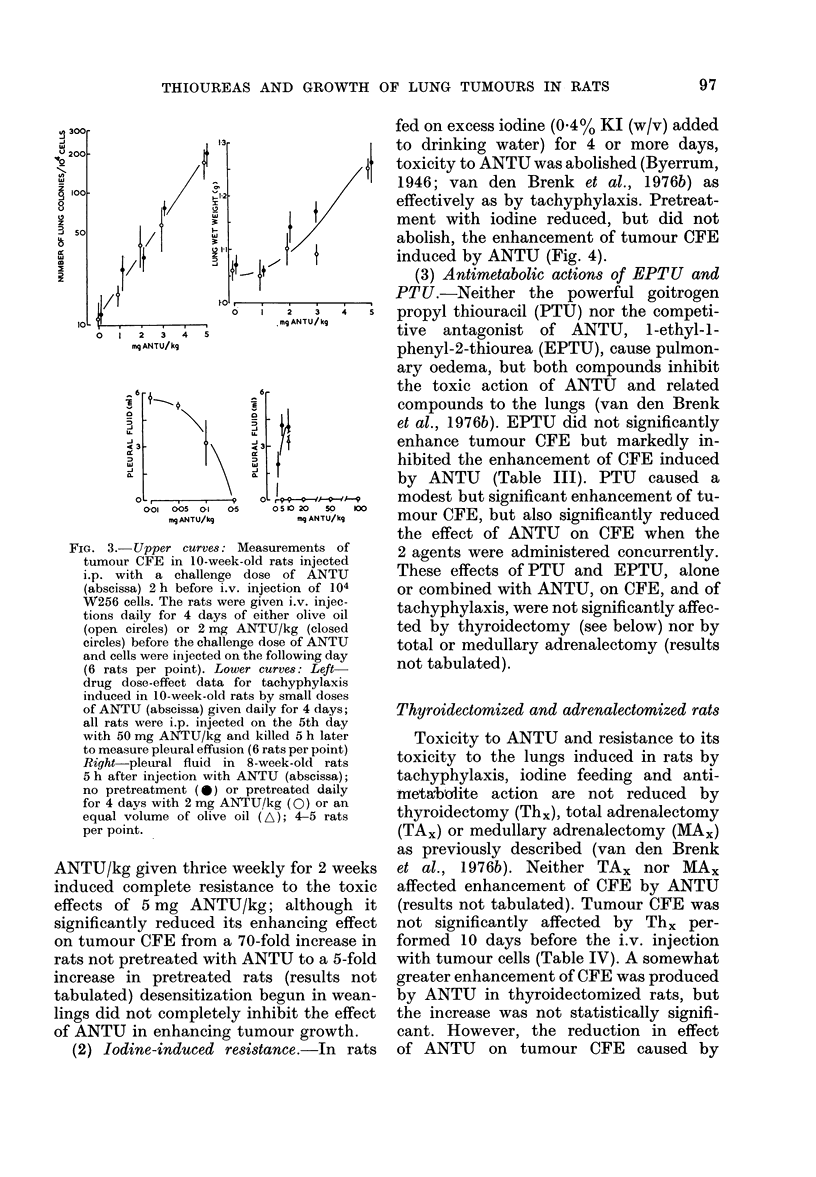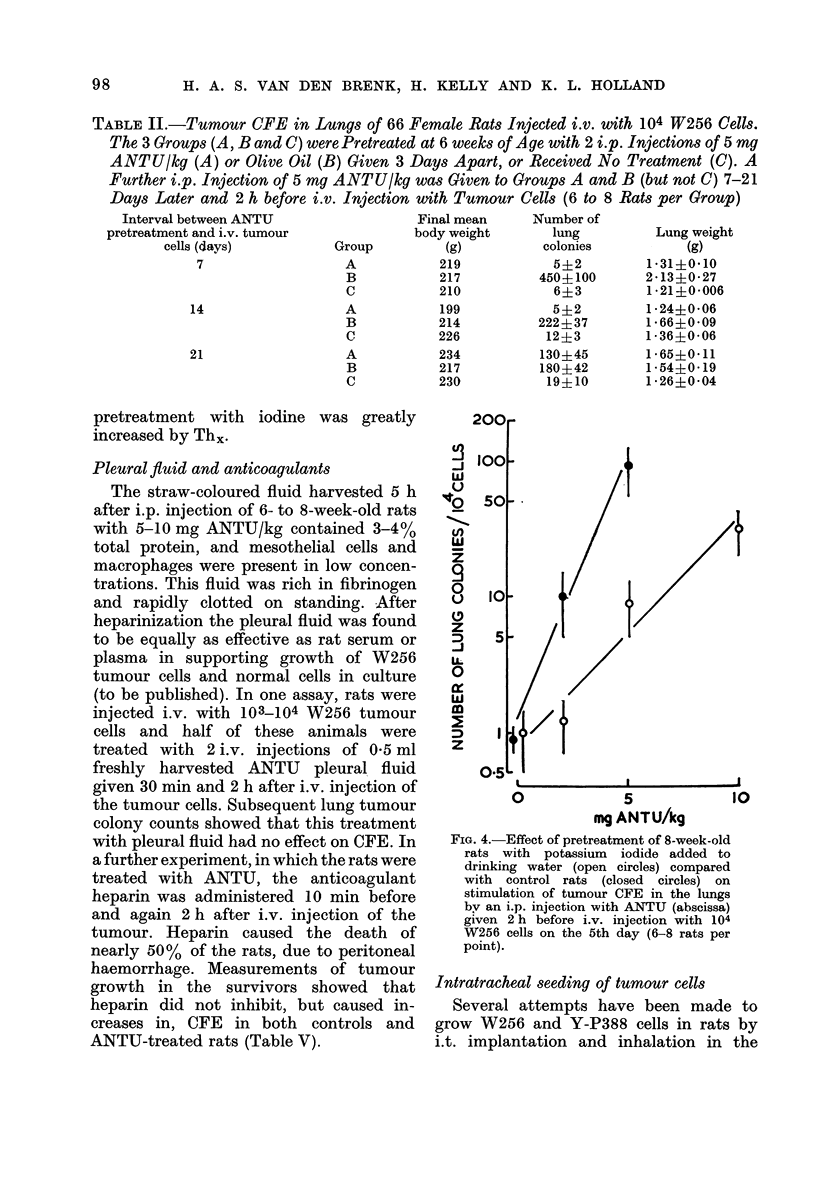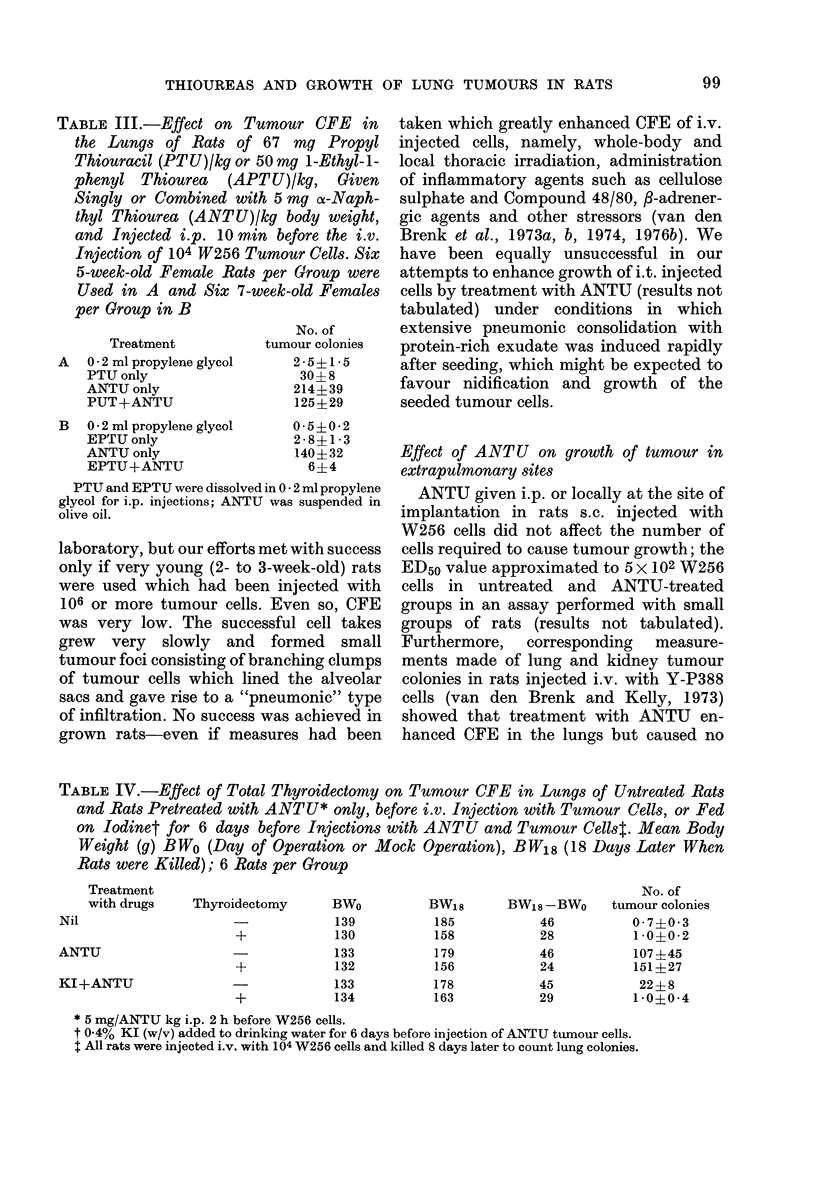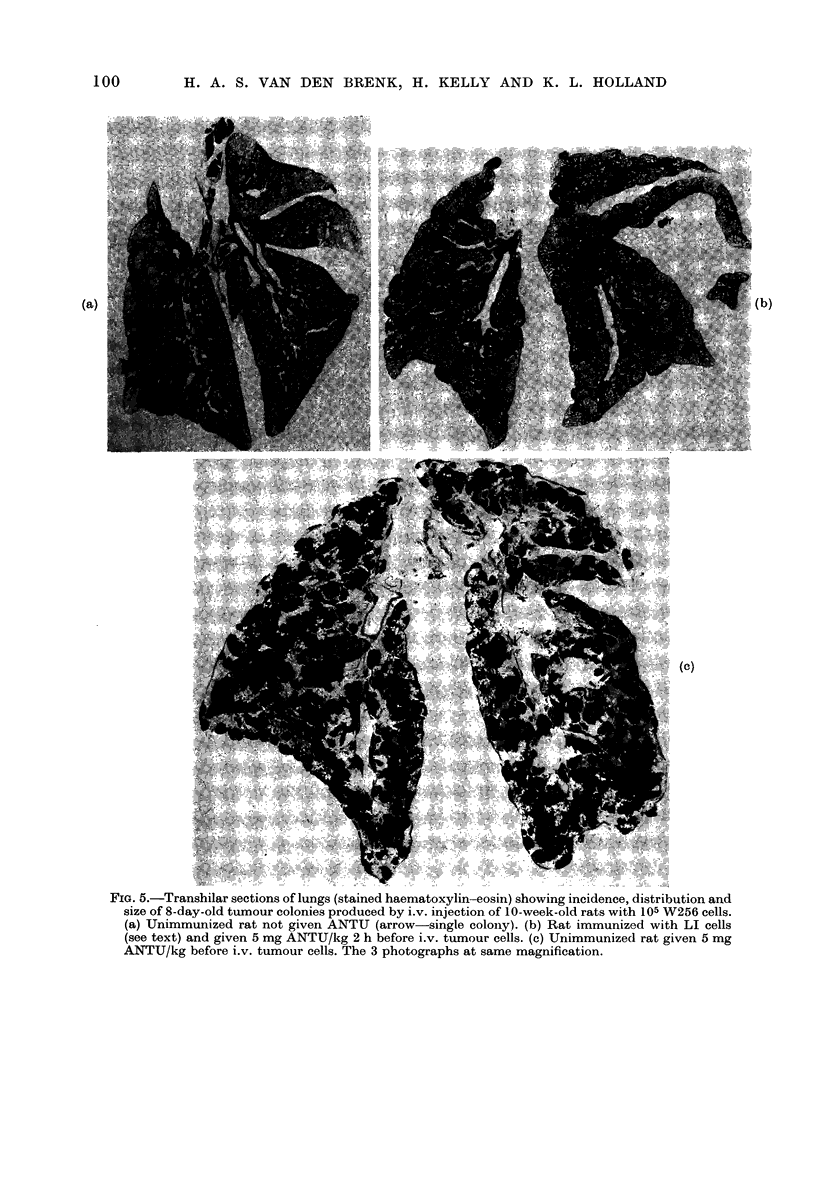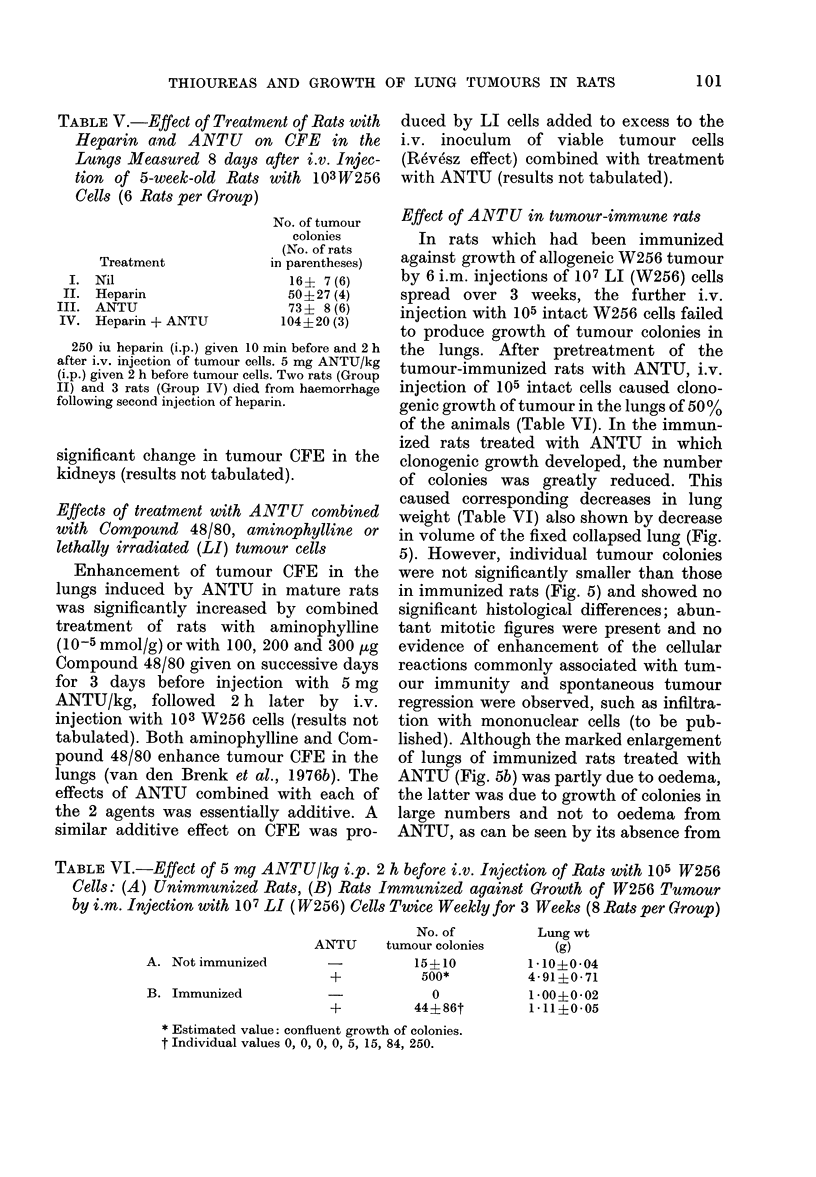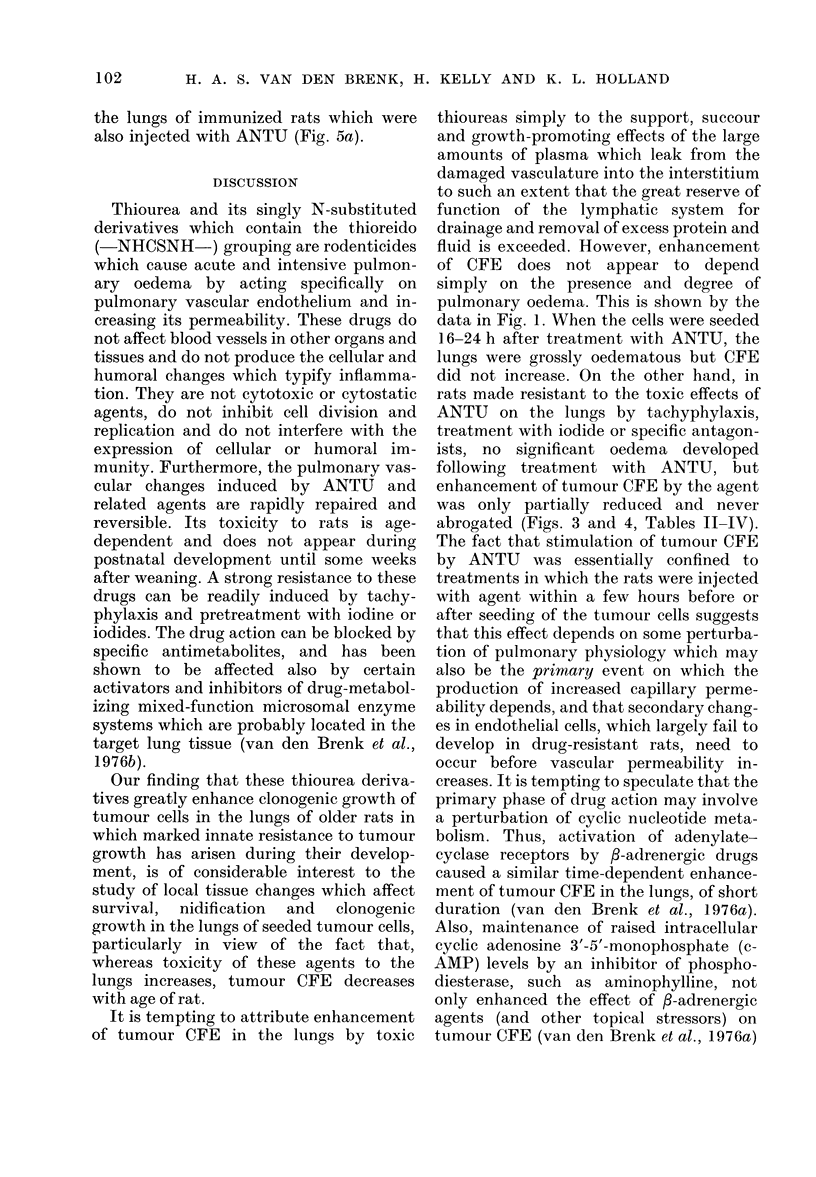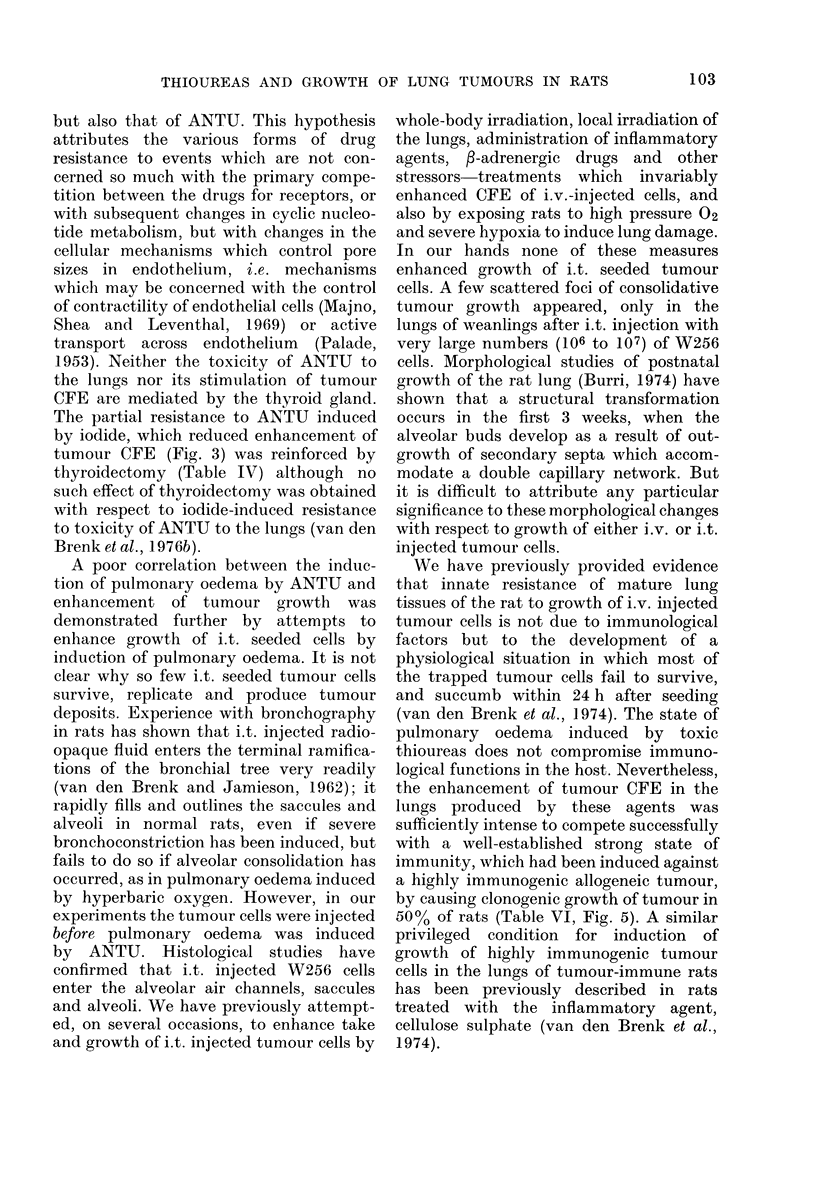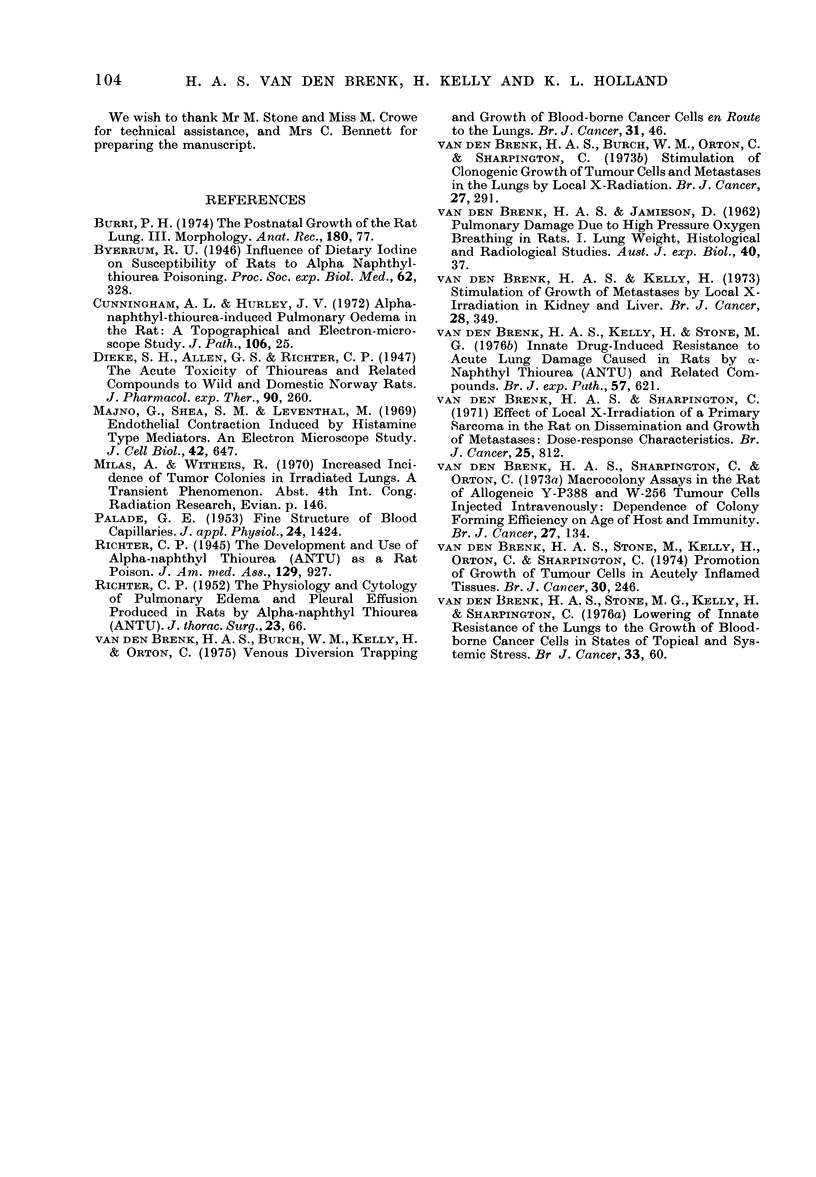# Effect of toxic thioureas on resistance of rats to growth in the lungs of intravenously and intratracheally seeded tumour cells.

**DOI:** 10.1038/bjc.1978.14

**Published:** 1978-01

**Authors:** H. A. van den Brenk, H. Kelly, K. L. Holland

## Abstract

**Images:**


*Br. J. Cancer* (1978) **37,** 92.

### EFFECT OF TOXIC THIOUREAS ON RESISTANCE OF RATS TO GROWTH IN THE LUNGS OF INTRAVENOUSLY AND INTRATRACHEALLY SEEDED TUMOUR CELLS

H. A. S. VAN DEN BRENK,* H. KELLY AND K. L. HOLLAND

*From the Richard Dimbleby Department of Cancer Research, St Thomas's Hospital Medical School, London SE1 7EH*

Received 20 June 1977 Accepted 16 August 1977

**Summary.—Clonogenic growth (colony-forming efficiency, CFE) of i.v. injected allogeneic W256 tumour cells in the lungs was markedly enhanced by treatment of rats with α-naphthyl thiourea (ANTU) injected i.p. from 2 h before to 2 h after the tumour cells. ANTU specifically increases pulmonary vascular permeability in adult rats and causes acute pulmonary oedema and pleural effusion. Inhibition of drug toxicity to the lungs by tachyphylaxis, specific antimetabolites or iodides did not abolish the effect of ANTU on CFE. CFE was not increased when cells were seeded by i.v. injection in lungs affected by advanced pulmonary oedema at 6 to 24 h after treatment with the drug. ANTU did not enhance growth of intratracheally injected cells.**

**Although ANTU has no cytotoxic or immunosuppressive action, treatment of tumour-immunized rats with ANTU caused apparent "breakdown" of tumour immunity in 50% of rats, by causing growth of tumour colonies in the lungs.**

**Possible mechanisms for the ANTU-induced decrease in innate resistance to growth of tumour in the lungs are discussed.**

INNATE susceptibility of the lungs to clonogenic growth of i.v. injected tumour cells decreases rapidly in the rat during the first 2 weeks after weaning (van den Brenk, Sharpington and Orton, 1973*a*). Susceptibility to tumour growth (measured in terms of tumour-colony-forming efficiency, CFE) in the lung and other organs, however, is markedly increased in grown rats by the induction of states of topical or systemic stress (van den Brenk *et al.*, 1976*a*). Thus, local X-irradiation of the lungs (van den Brenk *et al.*, 1973*b*; Milas and Withers, 1970) injection of rats with adrenergic drugs, inflammatory agents (cellulose sulphate, Compound 48/80) or chemical convulsants, and physical restraint of rats (van den Brenk *et al.*, 1974) markedly increased tumour CFE. These various stressors cause a perturbation of pulmonary physiology which appears to provide the *milieu propitieux* for survival and growth of seeded tumour cells. It seemed likely that the common disturbance in physiology caused by these stressors was an increase in permeability of the pulmonary exchange vessels, which enhanced tumour growth by allowing plasma, rich in growth-stimulating substances, to enter the extravascular compartment and enhance nidification of the seeded tumour cells.

In order to test this concept, we have studied the effects on tumour CFE in the lungs of treatment of rats with the rodenticide, α-naphthyl thiourea (ANTU), a compound which induces acute pulmonary oedema and pleural effusion (Richter, 1945) but has no significant effects on blood vessels and vascular permeability in other organs (Richter, 1952; Cunningham and Hurley, 1972). Related singly N-substituted derivatives of thiourea which contain the thioreido (—NHCSNH—) grouping show similar toxicity to rats (Dieke, Allen and Richter, 1947). These

* Present address: 64 Kooyong Road, Armadale, Victoria 3143, Australia.

compounds exhibit other pharmacological characteristics (van den Brenk, Kelly and Stone, 1976*b*) which add to their value as probes for studying tumour CFE in the lungs of rats. Thus, whereas weanling rats are highly resistant to their actions on the lungs, toxicity develops and increases rapidly during the 5th and 6th weeks of postnatal development, when innate resistance to tumour growth in the lungs develops. Also, marked resistance of mature rats to lung damage from toxic thioureas can be readily induced by tachyphylaxis and treatment with iodine and other agents. The toxicity to the lungs of ANTU and related toxic thioureas is not mediated by the thyroid or adrenal glands (van den Brenk *et al.*, 1976*b*); these agents do not inhibit cell replication, and have no significant effects on haemopoiesis or on immunological functions. The dosage of ANTU used to treat rats can be regulated to induce severe pulmonary oedema and effusion within a few hours, which rapidly resolves without causing lasting lung damage and allows rats to recover fully with 24–48 h. Advantage has been taken of these various aspects of the toxicology of ANTU and related compounds in studying and analysing their effects on tumour CFE in lungs of rats.

### MATERIALS AND METHODS

The techniques used in these experiments to determine and quantify CFE of i.v. injected tumour cells in the lungs of rats have been described previously (van den Brenk *et al.*, 1973*a*), together with the methods used to prepare lethally irradiated (LI) tumour cells, to irradiate locally the lungs of rats and to immunize rats against allogeneic Walker (W256) tumour. Female Carworth Farm rats of a specific pathogen-free (SPF)-derived colony were used which were singularly free from lung infection or other pulmonary disorders. The tumour was passaged as an ascites tumour and prepared as a cell suspension as previously described for injection of rats and quantitative bioassay of growth of tumour colonies in the lungs. The latter were counted 8 days after i.v. injection of tumour cells in the unfixed lungs of exsanguinated rats, when they were macroscopically visible as blood-red colonies, 1 to 2 mm in diameter.

The principal agent used (1-(1-naphthyl)-2-thiourea=α-naphthyl thiourea, ANTU, Koch Light Laboratories) was prepared as a suspension in olive oil or Mazola oil by sonication, for i.p. injection of rats. In a previous publication (van den Brenk *et al.*, 1976*b*) the sources of supply, preparation, administration and effects of other compounds used in these experiments are described; also the techniques used to perform thyroidectomy and adrenalectomy, the measurement of pulmonary oedema and pleural effusion induced by toxic thioureas, induction of tachyphylaxis, and the production of resistance to these drugs by the feeding of rats with potassium iodide, injection with specific antimetabolites or by treatment with agents which modify toxicity by stimulating the activities of drug-metabolizing microsomal enzymes. In this previous paper we published data concerning dosage and age-dependent toxicity of ANTU and related compounds in the same strain of rats as that used in the present experiments, and described the pharmacodynamic actions of ANTU in causing pulmonary oedema and the rate of its resolution. The design of the present experiments on tumour growth has been very largely influenced by these previous data, particularly in selecting time intervals between injection of rats with the drugs and tumour cells. However, in many of the tumour experiments, additional control groups of rats (injected with the agent(s) but not with tumour) were included, and killed at appropriate times to measure the incidence of pulmonary oedema and pleural effusion, and to determine the changes in histological appearances extant at the time of injection of the corresponding groups of rats with tumour cells.

*Intratracheal* (*i.t.*) *injection of tumour cells.*—Tumour cells were counted and dilutions of $10^6$ to $10^8$ cells per ml in Tyrode's solution (pH 7·3) were prepared for i.t. injection. Rats were lightly anaesthetized and the skin covering the anterior neck was shaved. This area was liberally swabbed with an antiseptic solution (0·5% chlorhexidine in alcohol) and a small longitudinal incision made through the skin of the lower neck overlying the cricoid and upper tracheal cartilages, which were exposed by deepening the incision through the pretracheal musculature. The required number of tumour cells contained in a volume of

0·1 ml was drawn up into a 1ml syringe, followed by an additional 0·5 ml of air. The contents of the syringe were then rapidly injected, at inspiration, into the upper trachea, entered immediately below the cricoid cartilage. The syringe was withdrawn and the skin wound was closed with a single suture. If the rat was cyanosed it was placed in a chamber aerated with 95% $O_2$/5% $CO_2$ to recover. It was then transferred to the X-ray therapy apparatus described previously (van den Brenk and Sharpington, 1971) and the whole of the lower portion of the neck (including the entire wound) was given a single dose of 1000 rad X-rays, taking care to shield the entire thorax and remainder of the body with 3mm-thick lead sheeting. This postoperative treatment of the neck following i.t. injection with tumour cells was mandatory; it prevented local recurrence of growth of solid tumour at the site of injection, which otherwise invariably occurs and causes asphyxiation within a week after i.t. injection of the cells. The lungs of i.t. injected rats were inspected for macroscopic evidence of tumour growth, and then placed in buffered neutral formalin for fixation and the preparation of serial sections stained with haematoxylin-eosin for histological examination.

FIG. 1.—Tumour CFE measurements in lungs of 7-week-old rats injected i.p. with 5 mg ANTU/kg (closed circles) at intervals before (negative values) or after i.v. injection with $10^4$ W256 tumour cells. Also shown are CFE values for rats injected with ANTU as weanlings and injected with cells only 52 days later, when aged 7 weeks (▲), or similarly treated weanlings given a second injection of ANTU immediately before cells at 7 weeks (△); 7-week-old rats injected with olive oil (□) or saline(○) only 2 h before cells (6 to 8 rats per point). The rate of production of pulmonary oedema (shown as measurements of volume of free pleural fluid) and its rate of resolution are shown for 7-week-old rats killed 0, 2, 5, 24 and 48 h after i.p. injection with 5 mg ANTU/kg (× dotted line, 5–8 rats per point).

### RESULTS

### *Tumour CFE in ANTU-treated rats*

(*1*) *Toxicity of ANTU*.—I.p. injection of grown rats, aged 6 weeks or more, with 5 mg ANTU/kg body wt caused acute pulmonary oedema and pleural effusion to appear 2–3 h after injection; the oedema rapidly increased and reached maximum intensity at about 4–5 h, and thereafter over the next 24–48 h it resolved slowly but completely (van den Brenk *et al.*, 1976*b*; see Fig. 1). It was found that over 90% of such older rats given 5 mg/kg survived, but that death usually occurred within 8 h when the dose was increased to 10 mg ANTU/kg. A smaller dose of 2 mg ANTU/kg rarely caused pulmonary oedema and pleural effusion. In young (3- to 4-week-old) rats, doses of 10–100 mg ANTU/kg failed to cause pulmonary oedema, but subsequently toxicity to the agent developed rapidly during the 5th and 6th weeks.

Older rats could be made highly resistant to the toxic effects of ANTU on the lungs by (1) tachyphylaxis, induced by injecting rats with small (non-toxic) doses of ANTU (or other thiourea derivatives) for a few days before challenge with larger doses of 5 mg/kg or more, (2) pretreatment for 4–5 days with iodine or KI added to their drinking water, (3) combined injection of ANTU with 1-ethyl-1-phenyl-2-thiourea (EPTU) or propyl thiouracil (PTU) which act as specific antimetabolites in antagonizing the toxicity of ANTU and related toxic thioureas to the lungs. These effects are described in detail in an earlier publication (van den Brenk *et al.*, 1976*b*) in which it was also shown that the pulmonary toxicity of ANTU and its inhibition by tachyphylaxis, iodides and antimetabolites such as EPTU and PTU, are not mediated by the thyroid gland and are not significantly affected by thyroidectomy or by treatment with thyroid or adrenocortical hormones or by adrenergic agonists. It is important to draw attention to our findings that tachyphylaxis could be induced at an early age in weanling rats, during a stage of development when the rat was highly resistant to the toxic effects of ANTU on the lungs, but sensitive to the goitrogenic effects of thiourea and other goitrogens. Such weanlings, pretreated with low or high doses of ANTU, or with thiourea, remained resistant for several weeks to a further challenge with ANTU in high dosage given when the rats would have otherwise developed toxicity to ANTU and related compounds.

(2) *Stimulation of CFE; drug dose-effect relationship.*—In young rats, innate resistance to growth of i.v. injected W256 cells in the lungs is low and tumour CFE is high (van den Brenk *et al.*, 1973*a*). Injection of weanling rats with ANTU in doses of 5 to 50 mg/kg caused no pulmonary oedema and only slight increases in tumour CFE when given 2–3 h before the tumour cells. Similar treatment of older (ANTU-sensitive) rats with a toxic dose of 4–5 mg ANTU/kg caused very marked increases in tumour CFE (Table I, Figs. 1 and 2). A non-toxic dose of 1 mg ANTU/kg had no significant effect, but CFE increased with increase in dose of ANTU (Fig. 2). Treatment of rats with the maximum tolerable dose (5 mg/kg) caused 100-fold or greater increases in CFE. The increases in lung weight measured 8 days after the injection of ANTU and tumour cells were largely due to the weight of growing lung tumour colonies; they were not due to generalized oedema induced by ANTU, which resolved within the first few days, irrespective of additional injections with tumour cells. ANTU did not significantly affect the weights of thymus and spleen in tumour-injected rats, and had no effect on the rate of body growth after the first 24–48 h (Fig. 2).

(3) *Times of injection of ANTU and tumour cells.*—CFE was not significantly affected in grown rats by 5 mg ANTU/kg injected 5 days before the tumour cells, but it progressively increased as this interval was shortened to 24 h and it increased even more steeply as the interval was decreased from 10 h to zero (Fig. 1).

Maximum increases in CFE were ob-

TABLE I.—*Effect of i.p. α-Naphthyl Thiourea (ANTU) 2 h before i.v. Injection with W256 Tumour Cells on Tumour CFE in the Lungs of Rats of Different Ages, Measured 8 days Later (6–8 Female Rats per Group)*

| Age (weeks) | Number of i.v. injected W256 cells | Dose ANTU (mg/kg) | Number of lung colonies | Increase in CFE (fold) | Lung weight (g) |
|---|---|---|---|---|---|
| 4 | $10^3$ | nil | 29±12 | — | 1·00±0·05 |
| | | 10 | 87±27 | 3 | 1·05±0·06 |
| 5 | $10^3$ | nil | 12±7 | — | 0·98±0·02 |
| | | 5 | 135±30 | 11 | 1·27±0·07 |
| 8 | $10^4$ | nil | 3±2 | — | 1·19±0·03 |
| | | 5 | 221±65 | 74 | 1·60±0·09 |
| 12 | $10^4$ | nil | 2±1 | — | 1·24±0·05 |
| | | 4 | 110±29 | 55 | 1·82±0·05 |
| | | 4* | 6±4 | 3 | 1·52±0·09 |

* ANTU injected 24 h *after* tumour cells.

FIG. 2.—Dose-effect curve (closed symbols) for tumour CFE in lungs of 8-week-old rats injected i.p. with single doses of ANTU (mg/kg) 2 h before i.v. injection with $10^4$ W256 tumour cells and killed 8 days later to count lung tumour colonies, organ wet weights and rate of body growth (ΔW/T g/day); in the 2 groups injected with tumour cells, no ANTU but an equal volume of saline (○) or olive oil (△) was injected (6 rats per group).

tained when ANTU was injected from −2 (before) to +2 h (after) i.v. injection of tumour cells. CFE rapidly decreased when ANTU was injected after the tumour cells, and no enhancement of CFE occurred when 16 h had elapsed before treatment. It is stressed that at no time was CFE enhanced by i.p. injection of rats with the vehicle (olive oil or Mazola oil) used to suspend ANTU for i.p. injections. The presence of pulmonary oedema in itself did not stimulate survival and growth of the seeded tumour cells, since no significant stimulation of CFE occurred when ANTU was injected at −24 to −5 h, so that the seeding of cells occurred when lung oedema and effusion were both well established. The maximum enhancement of CFE was induced by ANTU given 2 h before or after seeding of cells into lung tissue in which the oedematous change had not yet developed (Fig. 1). No enhancement of CFE occurred when ANTU was given to weanlings 52 days before the tumour cells were injected. This treatment of weanlings with a single large dose of ANTU induced tachyphylaxis which persisted for several weeks, so that the second dose of ANTU, given 2 h before injection with tumour cells at 11 weeks of age, did not enhance CFE (Fig. 1).

Trapping of i.v. injected tumour cells and distribution of tumour colonies were not affected by ANTU. The great majority of i.v. injected W256 tumour cells are trapped in the lungs (van den Brenk *et al.*, 1975); the aortic blood remains remarkably free of i.v. injected tumour cells, tumour colonies are confined to the lungs, and neither colonies nor microscopic evidence of tumour were detected in other organs when the lung colonies were counted 8 days or later after i.v. injection of the cells.

*Tumour growth in ANTU- and thiourea-resistant rats*

(1) *Tachyphylaxis.*—Pulmonary oedema and effusion produced in 6-week-old or older rats by ANTU could be virtually abolished by pretreatment of rats on 3 or 4 successive days with smaller doses (0·5 to 2 mg/kg) ANTU (see Fig. 3; van den Brenk *et al.*, 1976*b*). Toxicity could be similarly abolished by pretreatment with small doses of thiourea. However, rapid induction of tachyphylaxis in this way failed to affect significantly the enhancement of CFE by ANTU (Fig. 3). In further experiments it was found that rats which had recovered from pulmonary damage induced by toxic doses of ANTU, and in which a high degree of resistance to its toxic actions on the lungs persisted for several weeks, exhibited greater resistance to clonogenic growth of tumour cells. The resistance to tumour growth persisted for 14 days, but thereafter decreased when rats were retreated with ANTU (Table II), and despite the fact that a high level of resistance of the lungs to its toxic action persisted for at least another 3 weeks. Desensitization of weanlings with 2 mg

FIG. 3.—*Upper curves:* Measurements of tumour CFE in 10-week-old rats injected i.p. with a challenge dose of ANTU (abscissa) 2 h before i.v. injection of $10^4$ W256 cells. The rats were given i.v. injections daily for 4 days of either olive oil (open circles) or 2 mg ANTU/kg (closed circles) before the challenge dose of ANTU and cells were injected on the following day (6 rats per point). *Lower curves: Left*—drug dose-effect data for tachyphylaxis induced in 10-week-old rats by small doses of ANTU (abscissa) given daily for 4 days; all rats were i.p. injected on the 5th day with 50 mg ANTU/kg and killed 5 h later to measure pleural effusion (6 rats per point) *Right*—pleural fluid in 8-week-old rats 5 h after injection with ANTU (abscissa); no pretreatment (●) or pretreated daily for 4 days with 2 mg ANTU/kg (○) or an equal volume of olive oil (△); 4–5 rats per point.

ANTU/kg given thrice weekly for 2 weeks induced complete resistance to the toxic effects of 5 mg ANTU/kg; although it significantly reduced its enhancing effect on tumour CFE from a 70-fold increase in rats not pretreated with ANTU to a 5-fold increase in pretreated rats (results not tabulated) desensitization begun in weanlings did not completely inhibit the effect of ANTU in enhancing tumour growth.

(2) *Iodine-induced resistance.*—In rats fed on excess iodine (0·4% KI (w/v) added to drinking water) for 4 or more days, toxicity to ANTU was abolished (Byerrum, 1946; van den Brenk *et al.*, 1976*b*) as effectively as by tachyphylaxis. Pretreatment with iodine reduced, but did not abolish, the enhancement of tumour CFE induced by ANTU (Fig. 4).

(3) *Antimetabolic actions of EPTU and PTU.*—Neither the powerful goitrogen propyl thiouracil (PTU) nor the competitive antagonist of ANTU, 1-ethyl-1-phenyl-2-thiourea (EPTU), cause pulmonary oedema, but both compounds inhibit the toxic action of ANTU and related compounds to the lungs (van den Brenk *et al.*, 1976*b*). EPTU did not significantly enhance tumour CFE but markedly inhibited the enhancement of CFE induced by ANTU (Table III). PTU caused a modest but significant enhancement of tumour CFE, but also significantly reduced the effect of ANTU on CFE when the 2 agents were administered concurrently. These effects of PTU and EPTU, alone or combined with ANTU, on CFE, and of tachyphylaxis, were not significantly affected by thyroidectomy (see below) nor by total or medullary adrenalectomy (results not tabulated).

### *Thyroidectomized and adrenalectomized rats*

Toxicity to ANTU and resistance to its toxicity to the lungs induced in rats by tachyphylaxis, iodine feeding and antimetabolite action are not reduced by thyroidectomy ($Th_x$), total adrenalectomy ($TA_x$) or medullary adrenalectomy ($MA_x$) as previously described (van den Brenk *et al.*, 1976*b*). Neither $TA_x$ nor $MA_x$ affected enhancement of CFE by ANTU (results not tabulated). Tumour CFE was not significantly affected by $Th_x$ performed 10 days before the i.v. injection with tumour cells (Table IV). A somewhat greater enhancement of CFE was produced by ANTU in thyroidectomized rats, but the increase was not statistically significant. However, the reduction in effect of ANTU on tumour CFE caused by

TABLE II.—*Tumour CFE in Lungs of* 66 *Female Rats Injected i.v. with* $10^4$ *W*256 *Cells. The* 3 *Groups* (*A, B and C*) *were Pretreated at* 6 *weeks of Age with* 2 *i.p. Injections of* 5 *mg ANTU/kg* (*A*) *or Olive Oil* (*B*) *Given* 3 *Days Apart, or Received No Treatment* (*C*). *A Further i.p. Injection of* 5 *mg ANTU/kg was Given to Groups A and B* (*but not C*) 7–21 *Days Later and* 2 *h before i.v. Injection with Tumour Cells* (6 *to* 8 *Rats per Group*)

| Interval between ANTU pretreatment and i.v. tumour cells (days) | Group | Final mean body weight (g) | Number of lung colonies | Lung weight (g) |
|---|---|---|---|---|
| 7 | A | 219 | 5±2 | 1·31±0·10 |
| | B | 217 | 450±100 | 2·13±0·27 |
| | C | 210 | 6±3 | 1·21±0·006 |
| 14 | A | 199 | 5±2 | 1·24±0·06 |
| | B | 214 | 222±37 | 1·66±0·09 |
| | C | 226 | 12±3 | 1·36±0·06 |
| 21 | A | 234 | 130±45 | 1·65±0·11 |
| | B | 217 | 180±42 | 1·54±0·19 |
| | C | 230 | 19±10 | 1·26±0·04 |

pretreatment with iodine was greatly increased by $Th_X$.

*Pleural fluid and anticoagulants*

The straw-coloured fluid harvested 5 h after i.p. injection of 6- to 8-week-old rats with 5–10 mg ANTU/kg contained 3–4% total protein, and mesothelial cells and macrophages were present in low concentrations. This fluid was rich in fibrinogen and rapidly clotted on standing. After heparinization the pleural fluid was found to be equally as effective as rat serum or plasma in supporting growth of W256 tumour cells and normal cells in culture (to be published). In one assay, rats were injected i.v. with $10^3$–$10^4$ W256 tumour cells and half of these animals were treated with 2 i.v. injections of 0·5 ml freshly harvested ANTU pleural fluid given 30 min and 2 h after i.v. injection of the tumour cells. Subsequent lung tumour colony counts showed that this treatment with pleural fluid had no effect on CFE. In a further experiment, in which the rats were treated with ANTU, the anticoagulant heparin was administered 10 min before and again 2 h after i.v. injection of the tumour. Heparin caused the death of nearly 50% of the rats, due to peritoneal haemorrhage. Measurements of tumour growth in the survivors showed that heparin did not inhibit, but caused increases in, CFE in both controls and ANTU-treated rats (Table V).

FIG. 4.—Effect of pretreatment of 8-week-old rats with potassium iodide added to drinking water (open circles) compared with control rats (closed circles) on stimulation of tumour CFE in the lungs by an i.p. injection with ANTU (abscissa) given 2 h before i.v. injection with $10^4$ W256 cells on the 5th day (6–8 rats per point).

*Intratracheal seeding of tumour cells*

Several attempts have been made to grow W256 and Y-P388 cells in rats by i.t. implantation and inhalation in the

TABLE III.—*Effect on Tumour CFE in the Lungs of Rats of* 67 *mg Propyl Thiouracil (PTU)/kg or* 50 *mg* 1-*Ethyl*-1-*phenyl Thiourea (APTU)/kg, Given Singly or Combined with* 5 *mg α-Naphthyl Thiourea (ANTU)/kg body weight, and Injected i.p.* 10 *min before the i.v. Injection of* $10^4$ *W256 Tumour Cells. Six* 5-*week-old Female Rats per Group were Used in A and Six* 7-*week-old Females per Group in B*

| | Treatment | No. of tumour colonies |
|---|---|---|
| A | 0·2 ml propylene glycol | 2·5±1·5 |
| | PTU only | 30±8 |
| | ANTU only | 214±39 |
| | PUT+ANTU | 125±29 |
| B | 0·2 ml propylene glycol | 0·5±0·2 |
| | EPTU only | 2·8±1·3 |
| | ANTU only | 140±32 |
| | EPTU+ANTU | 6±4 |

PTU and EPTU were dissolved in 0·2 ml propylene glycol for i.p. injections; ANTU was suspended in olive oil.

laboratory, but our efforts met with success only if very young (2- to 3-week-old) rats were used which had been injected with $10^6$ or more tumour cells. Even so, CFE was very low. The successful cell takes grew very slowly and formed small tumour foci consisting of branching clumps of tumour cells which lined the alveolar sacs and gave rise to a "pneumonic" type of infiltration. No success was achieved in grown rats—even if measures had been taken which greatly enhanced CFE of i.v. injected cells, namely, whole-body and local thoracic irradiation, administration of inflammatory agents such as cellulose sulphate and Compound 48/80, β-adrenergic agents and other stressors (van den Brenk *et al.*, 1973*a*, *b*, 1974, 1976*b*). We have been equally unsuccessful in our attempts to enhance growth of i.t. injected cells by treatment with ANTU (results not tabulated) under conditions in which extensive pneumonic consolidation with protein-rich exudate was induced rapidly after seeding, which might be expected to favour nidification and growth of the seeded tumour cells.

*Effect of ANTU on growth of tumour in extrapulmonary sites*

ANTU given i.p. or locally at the site of implantation in rats s.c. injected with W256 cells did not affect the number of cells required to cause tumour growth; the $ED_{50}$ value approximated to $5 \times 10^2$ W256 cells in untreated and ANTU-treated groups in an assay performed with small groups of rats (results not tabulated). Furthermore, corresponding measurements made of lung and kidney tumour colonies in rats injected i.v. with Y-P388 cells (van den Brenk and Kelly, 1973) showed that treatment with ANTU enhanced CFE in the lungs but caused no

TABLE IV.—*Effect of Total Thyroidectomy on Tumour CFE in Lungs of Untreated Rats and Rats Pretreated with ANTU* only, before i.v. Injection with Tumour Cells, or Fed on Iodine† for* 6 *days before Injections with ANTU and Tumour Cells‡. Mean Body Weight (g)* $BW_0$ *(Day of Operation or Mock Operation),* $BW_{18}$ *(*18 *Days Later When Rats were Killed);* 6 *Rats per Group*

| Treatment with drugs | Thyroidectomy | $BW_0$ | $BW_{18}$ | $BW_{18}-BW_0$ | No. of tumour colonies |
|---|---|---|---|---|---|
| Nil | — | 139 | 185 | 46 | 0·7±0·3 |
| | + | 130 | 158 | 28 | 1·0±0·2 |
| ANTU | — | 133 | 179 | 46 | 107±45 |
| | + | 132 | 156 | 24 | 151±27 |
| KI+ANTU | — | 133 | 178 | 45 | 22±8 |
| | + | 134 | 163 | 29 | 1·0±0·4 |

* 5 mg/ANTU kg i.p. 2 h before W256 cells.
† 0·4% KI (w/v) added to drinking water for 6 days before injection of ANTU tumour cells.
‡ All rats were injected i.v. with $10^4$ W256 cells and killed 8 days later to count lung colonies.

FIG. 5.—Transhilar sections of lungs (stained haematoxylin–eosin) showing incidence, distribution and size of 8-day-old tumour colonies produced by i.v. injection of 10-week-old rats with $10^5$ W256 cells. (a) Unimmunized rat not given ANTU (arrow—single colony). (b) Rat immunized with LI cells (see text) and given 5 mg ANTU/kg 2 h before i.v. tumour cells. (c) Unimmunized rat given 5 mg ANTU/kg before i.v. tumour cells. The 3 photographs at same magnification.

TABLE V.—*Effect of Treatment of Rats with Heparin and ANTU on CFE in the Lungs Measured 8 days after i.v. Injection of 5-week-old Rats with $10^3$ W256 Cells (6 Rats per Group)*

| | Treatment | No. of tumour colonies (No. of rats in parentheses) |
|---|---|---|
| I. | Nil | 16± 7 (6) |
| II. | Heparin | 50±27 (4) |
| III. | ANTU | 73± 8 (6) |
| IV. | Heparin + ANTU | 104±20 (3) |

250 iu heparin (i.p.) given 10 min before and 2 h after i.v. injection of tumour cells. 5 mg ANTU/kg (i.p.) given 2 h before tumour cells. Two rats (Group II) and 3 rats (Group IV) died from haemorrhage following second injection of heparin.

significant change in tumour CFE in the kidneys (results not tabulated).

*Effects of treatment with ANTU combined with Compound 48/80, aminophylline or lethally irradiated (LI) tumour cells*

Enhancement of tumour CFE in the lungs induced by ANTU in mature rats was significantly increased by combined treatment of rats with aminophylline ($10^{-5}$ mmol/g) or with 100, 200 and 300 μg Compound 48/80 given on successive days for 3 days before injection with 5 mg ANTU/kg, followed 2 h later by i.v. injection with $10^3$ W256 cells (results not tabulated). Both aminophylline and Compound 48/80 enhance tumour CFE in the lungs (van den Brenk *et al.*, 1976*b*). The effects of ANTU combined with each of the 2 agents was essentially additive. A similar additive effect on CFE was produced by LI cells added to excess to the i.v. inoculum of viable tumour cells (Révész effect) combined with treatment with ANTU (results not tabulated).

*Effect of ANTU in tumour-immune rats*

In rats which had been immunized against growth of allogeneic W256 tumour by 6 i.m. injections of $10^7$ LI (W256) cells spread over 3 weeks, the further i.v. injection with $10^5$ intact W256 cells failed to produce growth of tumour colonies in the lungs. After pretreatment of the tumour-immunized rats with ANTU, i.v. injection of $10^5$ intact cells caused clonogenic growth of tumour in the lungs of 50% of the animals (Table VI). In the immunized rats treated with ANTU in which clonogenic growth developed, the number of colonies was greatly reduced. This caused corresponding decreases in lung weight (Table VI) also shown by decrease in volume of the fixed collapsed lung (Fig. 5). However, individual tumour colonies were not significantly smaller than those in immunized rats (Fig. 5) and showed no significant histological differences; abundant mitotic figures were present and no evidence of enhancement of the cellular reactions commonly associated with tumour immunity and spontaneous tumour regression were observed, such as infiltration with mononuclear cells (to be published). Although the marked enlargement of lungs of immunized rats treated with ANTU (Fig. 5*b*) was partly due to oedema, the latter was due to growth of colonies in large numbers and not to oedema from ANTU, as can be seen by its absence from

TABLE VI.—*Effect of 5 mg ANTU/kg i.p. 2 h before i.v. Injection of Rats with $10^5$ W256 Cells: (A) Unimmunized Rats, (B) Rats Immunized against Growth of W256 Tumour by i.m. Injection with $10^7$ LI (W256) Cells Twice Weekly for 3 Weeks (8 Rats per Group)*

| | ANTU | No. of tumour colonies | Lung wt (g) |
|---|---|---|---|
| A. Not immunized | — | 15±10 | 1·10±0·04 |
| | + | 500* | 4·91±0·71 |
| B. Immunized | — | 0 | 1·00±0·02 |
| | + | 44±86† | 1·11±0·05 |

* Estimated value: confluent growth of colonies.
† Individual values 0, 0, 0, 0, 5, 15, 84, 250.

the lungs of immunized rats which were also injected with ANTU (Fig. 5*a*).

### DISCUSSION

Thiourea and its singly N-substituted derivatives which contain the thioreido (—NHCSNH—) grouping are rodenticides which cause acute and intensive pulmonary oedema by acting specifically on pulmonary vascular endothelium and increasing its permeability. These drugs do not affect blood vessels in other organs and tissues and do not produce the cellular and humoral changes which typify inflammation. They are not cytotoxic or cytostatic agents, do not inhibit cell division and replication and do not interfere with the expression of cellular or humoral immunity. Furthermore, the pulmonary vascular changes induced by ANTU and related agents are rapidly repaired and reversible. Its toxicity to rats is age-dependent and does not appear during postnatal development until some weeks after weaning. A strong resistance to these drugs can be readily induced by tachyphylaxis and pretreatment with iodine or iodides. The drug action can be blocked by specific antimetabolites, and has been shown to be affected also by certain activators and inhibitors of drug-metabolizing mixed-function microsomal enzyme systems which are probably located in the target lung tissue (van den Brenk *et al.*, 1976*b*).

Our finding that these thiourea derivatives greatly enhance clonogenic growth of tumour cells in the lungs of older rats in which marked innate resistance to tumour growth has arisen during their development, is of considerable interest to the study of local tissue changes which affect survival, nidification and clonogenic growth in the lungs of seeded tumour cells, particularly in view of the fact that, whereas toxicity of these agents to the lungs increases, tumour CFE decreases with age of rat.

It is tempting to attribute enhancement of tumour CFE in the lungs by toxic thioureas simply to the support, succour and growth-promoting effects of the large amounts of plasma which leak from the damaged vasculature into the interstitium to such an extent that the great reserve of function of the lymphatic system for drainage and removal of excess protein and fluid is exceeded. However, enhancement of CFE does not appear to depend simply on the presence and degree of pulmonary oedema. This is shown by the data in Fig. 1. When the cells were seeded 16–24 h after treatment with ANTU, the lungs were grossly oedematous but CFE did not increase. On the other hand, in rats made resistant to the toxic effects of ANTU on the lungs by tachyphylaxis, treatment with iodide or specific antagonists, no significant oedema developed following treatment with ANTU, but enhancement of tumour CFE by the agent was only partially reduced and never abrogated (Figs. 3 and 4, Tables II–IV). The fact that stimulation of tumour CFE by ANTU was essentially confined to treatments in which the rats were injected with agent within a few hours before or after seeding of the tumour cells suggests that this effect depends on some perturbation of pulmonary physiology which may also be the *primary* event on which the production of increased capillary permeability depends, and that secondary changes in endothelial cells, which largely fail to develop in drug-resistant rats, need to occur before vascular permeability increases. It is tempting to speculate that the primary phase of drug action may involve a perturbation of cyclic nucleotide metabolism. Thus, activation of adenylate–cyclase receptors by $\beta$-adrenergic drugs caused a similar time-dependent enhancement of tumour CFE in the lungs, of short duration (van den Brenk *et al.*, 1976*a*). Also, maintenance of raised intracellular cyclic adenosine 3′-5′-monophosphate (c-AMP) levels by an inhibitor of phosphodiesterase, such as aminophylline, not only enhanced the effect of $\beta$-adrenergic agents (and other topical stressors) on tumour CFE (van den Brenk *et al.*, 1976*a*)

but also that of ANTU. This hypothesis attributes the various forms of drug resistance to events which are not concerned so much with the primary competition between the drugs for receptors, or with subsequent changes in cyclic nucleotide metabolism, but with changes in the cellular mechanisms which control pore sizes in endothelium, *i.e.* mechanisms which may be concerned with the control of contractility of endothelial cells (Majno, Shea and Leventhal, 1969) or active transport across endothelium (Palade, 1953). Neither the toxicity of ANTU to the lungs nor its stimulation of tumour CFE are mediated by the thyroid gland. The partial resistance to ANTU induced by iodide, which reduced enhancement of tumour CFE (Fig. 3) was reinforced by thyroidectomy (Table IV) although no such effect of thyroidectomy was obtained with respect to iodide-induced resistance to toxicity of ANTU to the lungs (van den Brenk *et al.*, 1976*b*).

A poor correlation between the induction of pulmonary oedema by ANTU and enhancement of tumour growth was demonstrated further by attempts to enhance growth of i.t. seeded cells by induction of pulmonary oedema. It is not clear why so few i.t. seeded tumour cells survive, replicate and produce tumour deposits. Experience with bronchography in rats has shown that i.t. injected radio-opaque fluid enters the terminal ramifications of the bronchial tree very readily (van den Brenk and Jamieson, 1962); it rapidly fills and outlines the saccules and alveoli in normal rats, even if severe bronchoconstriction has been induced, but fails to do so if alveolar consolidation has occurred, as in pulmonary oedema induced by hyperbaric oxygen. However, in our experiments the tumour cells were injected *before* pulmonary oedema was induced by ANTU. Histological studies have confirmed that i.t. injected W256 cells enter the alveolar air channels, saccules and alveoli. We have previously attempted, on several occasions, to enhance take and growth of i.t. injected tumour cells by whole-body irradiation, local irradiation of the lungs, administration of inflammatory agents, $\beta$-adrenergic drugs and other stressors—treatments which invariably enhanced CFE of i.v.-injected cells, and also by exposing rats to high pressure $O_2$ and severe hypoxia to induce lung damage. In our hands none of these measures enhanced growth of i.t. seeded tumour cells. A few scattered foci of consolidative tumour growth appeared, only in the lungs of weanlings after i.t. injection with very large numbers ($10^6$ to $10^7$) of W256 cells. Morphological studies of postnatal growth of the rat lung (Burri, 1974) have shown that a structural transformation occurs in the first 3 weeks, when the alveolar buds develop as a result of outgrowth of secondary septa which accommodate a double capillary network. But it is difficult to attribute any particular significance to these morphological changes with respect to growth of either i.v. or i.t. injected tumour cells.

We have previously provided evidence that innate resistance of mature lung tissues of the rat to growth of i.v. injected tumour cells is not due to immunological factors but to the development of a physiological situation in which most of the trapped tumour cells fail to survive, and succumb within 24 h after seeding (van den Brenk *et al.*, 1974). The state of pulmonary oedema induced by toxic thioureas does not compromise immunological functions in the host. Nevertheless, the enhancement of tumour CFE in the lungs produced by these agents was sufficiently intense to compete successfully with a well-established strong state of immunity, which had been induced against a highly immunogenic allogeneic tumour, by causing clonogenic growth of tumour in 50% of rats (Table VI, Fig. 5). A similar privileged condition for induction of growth of highly immunogenic tumour cells in the lungs of tumour-immune rats has been previously described in rats treated with the inflammatory agent, cellulose sulphate (van den Brenk *et al.*, 1974).

We wish to thank Mr M. Stone and Miss M. Crowe for technical assistance, and Mrs C. Bennett for preparing the manuscript.

### REFERENCES

BURRI, P. H. (1974) The Postnatal Growth of the Rat Lung. III. Morphology. *Anat. Rec.*, **180**, 77.

BYERRUM, R. U. (1946) Influence of Dietary Iodine on Susceptibility of Rats to Alpha Naphthylthiourea Poisoning. *Proc. Soc. exp. Biol. Med.*, **62**, 328.

CUNNINGHAM, A. L. & HURLEY, J. V. (1972) Alphanaphthyl-thiourea-induced Pulmonary Oedema in the Rat: A Topographical and Electron-microscope Study. *J. Path.*, **106**, 25.

DIEKE, S. H., ALLEN, G. S. & RICHTER, C. P. (1947) The Acute Toxicity of Thioureas and Related Compounds to Wild and Domestic Norway Rats. *J. Pharmacol. exp. Ther.*, **90**, 260.

MAJNO, G., SHEA, S. M. & LEVENTHAL, M. (1969) Endothelial Contraction Induced by Histamine Type Mediators. An Electron Microscope Study. *J. Cell Biol.*, **42**, 647.

MILAS, A. & WITHERS, R. (1970) Increased Incidence of Tumor Colonies in Irradiated Lungs. A Transient Phenomenon. Abst. 4th Int. Cong. Radiation Research, Evian. p. 146.

PALADE, G. E. (1953) Fine Structure of Blood Capillaries. *J. appl. Physiol.*, **24**, 1424.

RICHTER, C. P. (1945) The Development and Use of Alpha-naphthyl Thiourea (ANTU) as a Rat Poison. *J. Am. med. Ass.*, **129**, 927.

RICHTER, C. P. (1952) The Physiology and Cytology of Pulmonary Edema and Pleural Effusion Produced in Rats by Alpha-naphthyl Thiourea (ANTU). *J. thorac. Surg.*, **23**, 66.

VAN DEN BRENK, H. A. S., BURCH, W. M., KELLY, H. & ORTON, C. (1975) Venous Diversion Trapping and Growth of Blood-borne Cancer Cells *en Route* to the Lungs. *Br. J. Cancer*, **31**, 46.

VAN DEN BRENK, H. A. S., BURCH, W. M., ORTON, C. & SHARPINGTON, C. (1973*b*) Stimulation of Clonogenic Growth of Tumour Cells and Metastases in the Lungs by Local X-Radiation. *Br. J. Cancer*, **27**, 291.

VAN DEN BRENK, H. A. S. & JAMIESON, D. (1962) Pulmonary Damage Due to High Pressure Oxygen Breathing in Rats. I. Lung Weight, Histological and Radiological Studies. *Aust. J. exp. Biol.*, **40**, 37.

VAN DEN BRENK, H. A. S. & KELLY, H. (1973) Stimulation of Growth of Metastases by Local X-Irradiation in Kidney and Liver. *Br. J. Cancer*, **28**, 349.

VAN DEN BRENK, H. A. S., KELLY, H. & STONE, M. G. (1976*b*) Innate Drug-Induced Resistance to Acute Lung Damage Caused in Rats by α-Naphthyl Thiourea (ANTU) and Related Compounds. *Br. J. exp. Path.*, **57**, 621.

VAN DEN BRENK, H. A. S. & SHARPINGTON, C. (1971) Effect of Local X-Irradiation of a Primary Sarcoma in the Rat on Dissemination and Growth of Metastases: Dose-response Characteristics. *Br. J. Cancer*, **25**, 812.

VAN DEN BRENK, H. A. S., SHARPINGTON, C. & ORTON, C. (1973*a*) Macrocolony Assays in the Rat of Allogeneic Y-P388 and W-256 Tumour Cells Injected Intravenously: Dependence of Colony Forming Efficiency on Age of Host and Immunity. *Br. J. Cancer*, **27**, 134.

VAN DEN BRENK, H. A. S., STONE, M., KELLY, H., ORTON, C. & SHARPINGTON, C. (1974) Promotion of Growth of Tumour Cells in Acutely Inflamed Tissues. *Br. J. Cancer*, **30**, 246.

VAN DEN BRENK, H. A. S., STONE, M. G., KELLY, H. & SHARPINGTON, C. (1976*a*) Lowering of Innate Resistance of the Lungs to the Growth of Blood-borne Cancer Cells in States of Topical and Systemic Stress. *Br J. Cancer*, **33**, 60.